# When a Calling Goes Unanswered: Exploring the Role of Workplace Personalizations as Calling Enactments

**DOI:** 10.3389/fpsyg.2019.01940

**Published:** 2019-09-13

**Authors:** Bruno Felix, Flavia Cavazotte

**Affiliations:** ^1^Fucape Business School, Vitória, Brazil; ^2^Pontifical Catholic University of Rio de Janeiro, Rio de Janeiro, Brazil

**Keywords:** unanswered callings, calling enactment, workplace personalization, foregone identities, calling symbols

## Abstract

Individuals are sometimes unable to realize their callings in their formal careers. The literature has highlighted that such unanswered callings produce negative outcomes in the individual’s career and personal life and that coping strategies, such as job and leisure crafting, can help them buffer such consequences. We developed a grounded theory regarding how people cope with their unanswered callings through a previously unexplored strategy in the calling literature: workplace personalization. Our study revealed that through this strategy, individuals retain the aspects of an unanswered calling in their self-concept and then reduce the consequences of not realizing the calling. Some participants enjoy some of the benefits of perceiving a calling, even without performing it in a formal work role. This phenomenon occurs because workplace personalization can be used to represent unanswered callings performed in the past and present, or that are intended to be performed in the future. This form of enactment produces interpersonal and intrapersonal processes that help buffer the negative consequences of not realizing a calling.

## Introduction

“I already understand the idea that, for now, I will not be able to live my calling as a teacher for children with special needs. But these handprints of my daughter in this frame help me remember myself and show others what my calling is (…). And it makes me feel better about myself. Somehow, it makes me feel that this calling is alive” (E12, woman, IT director).

The multiple career options available today along with the idea that people can find meaning, purpose, and opportunities to serve others through their work (Thompson and Bunderson, 2003; Wrzesniewski, 2003; Grant, 2007) are trends that have created a double-edged sword for workers. On the one hand, individuals often perceive that they are called to an occupation; this creates the expectation that work will be a domain of duties and obligations but also a source of pleasure and satisfaction through self-expression. On the other hand, due to practical limitations, many individuals will not perform these callings in a formal work role (Berg et al., 2010); consequently, they might experience undesired outcomes, and often show lower job and life satisfaction than those who are living their perceived calling or who do not have a calling (Gazica and Spector, 2015). This discrepancy is particularly relevant in the context of economic crises, when trends such as staff reduction and unemployment further prevent several individuals from living their callings, also provoking feelings of fear, anxiety, and depression (Giorgi et al., 2015; Mucci et al., 2016).

Are people who have unanswered callings condemned to frustration? Are there self-help strategies that can allow them to enact such callings in their lives? Berg et al. (2010) have investigated these question, focusing on job and leisure crafting as coping strategies. They observed that these strategies could promote meaning and enjoyment among individuals with unanswered callings. However, the authors also observed that such strategies can generate feelings of stress and regret over the forgone accomplishments of their unanswered callings. In the pursuit to answer their callings through job and leisure crafting, they also face the risk that these coping strategies would be seen as a sign of misfit or maladaptation to the current job. Are there other coping strategies that individuals with unanswered callings use to enact them without experiencing such negative consequences?

Recent developments in identity research suggest that people can enact an identity-related calling even when they don’t perform it in their formal work; individuals also enact their forgone identities through remembering their past or creating images of their future (Obodaru, 2017). Also, the literature on workplace personalization indicates that displaying symbolic objects at work can be a means of communicating desired aspects of one’s life to others without exposing them to too many conflicts (Goffman, 1959; Pratt and Rafaeli, 2001). Therefore, based on the ideas that callings can be enacted through imaginative processes and that the use of symbolic objects can communicate relevant information about the self, in this study we explore how individuals use workplace personalization to enact their unanswered callings. We argue that such calling symbols can allow individuals to enact their callings and buffer the negative consequences of having their callings unanswered.

Notably, the literature on callings has not yet explored the role of symbolic self-representations in the workplace, such as photos, diplomas, and other personal objects, as a means to represent and enact unanswered callings. We contribute to this literature in three ways: (a) we expand our understanding about calling enactment, by observing that it can take place even when callings are not performed in formal work roles; (b) we broaden knowledge regarding coping strategies used to manage unanswered callings, by discussing how calling symbols help individuals retain in their self-definitions aspects associated with their unanswered callings; and (c) we highlight that symbols of past, present, and future callings allow individuals to enact their unanswered callings while cutting down the risks of conflict with current work roles. This paper also contributes to identity research by unveiling that calling symbols used in workplace personalization induce interpersonal and intrapersonal processes that help individuals cope with their foregone identities.

## Literature Review

### Pursuit of Unanswered Callings: What Do We Know So Far?

A calling is an occupation that an individual is drawn to pursue, interprets as intrinsically enjoyable and meaningful, and views as a central part of their identity (Dobrow and Tosti-Kharas, 2010). The growing importance of callings in the career literature derives from studies on positive psychological health (Duffy and Sedlacek, 2010; Duffy et al., 2011c) and job-related outcomes (Cardador et al., 2011; Duffy et al., 2011a, 2012; Hirschi, 2012; Neubert and Halbesleben, 2015). However, recent evidence has suggested that the positive outcomes of having a calling are experienced only by those who live up their calling (Duffy et al., 2012; Gazica and Spector, 2015). When individuals cannot answer their callings, they tend to present poorer psychological health, and report lower life and job satisfaction (Duffy et al., 2011b, 2016). Such findings have stimulated discussions about the distinction between perceiving and living a calling (Duffy and Autin, 2013) and about unanswered callings.

An unanswered calling is a calling that an individual perceives but is not currently pursuing; thus, it is an attitude regarding an occupation that is not part of the individual’s occupational role (Berg et al., 2010). Studies on unanswered callings have often explored the negative consequences of not living a perceived calling. For example, Duffy et al. (2012) found that living a calling moderates the relationship between having a calling and career commitment, such that this relationship is weaker for individuals with unanswered callings. Duffy et al. (2011b) suggested that those who report a calling and the intention to withdraw from work may also have a calling they are not presently pursuing. Gazica and Spector (2015) observed that individuals with an unanswered calling reported more negative health- and work-related outcomes than those who did not have a calling and who had answered their callings.

The distinction between perceiving and living a calling (Duffy and Autin, 2013) has led to the understanding that a calling is “not just an abstract perception of oneself but rather a continual process of habitual activities that confers a sense of structure and a sense of coherence on one’s day life” (Thatcher and Zhu, 2006, p. 1077). According to Conway et al. (2015), a calling generates positive outcomes for individuals only when they engage in “calling enactment:” the more they perform activities that represent their calling, the more they enact their calling. This view conceives calling enactment as an activity restricted to an individual’s formal work domain; however, this view can be problematic because individuals sometimes enact a calling in activities beyond such boundaries. For example, Berg et al. (2010) described the role of job crafting (enacting a calling by performing activities that express this calling) and leisure crafting (enacting a calling during leisure time) as strategies that sooth the negative consequences of not living out a perceived calling. Job crafting occurs within the limits of a formal work role, and leisure crafting occurs in an individual’s private time and space. Thus, a question arises: Are there any strategies other than job and leisure crafting that individuals can use to answer their callings? In the section “Identity as a Lens to Reveal How People Cope With Unanswered Callings,” we draw on findings from identity research to highlight the sensitizing concepts that sustain our research.

### Identity as a Lens to Reveal How People Cope With Unanswered Callings

As the definition of callings adopted in this study conceives it is a central part of an individual’s identity (Dobrow and Tosti-Kharas, 2010), we apply developments from identity research to elucidate how individuals cope with the negative consequences of not living a perceived calling. More specifically, we focus on the concept of identity enactment to establish a parallel with the concept of calling enactment (Conway et al., 2015; Lysova and Khapova, 2018).

“Identity enactment” is the process of “acting out” or claiming an identity by engaging in behaviors associated with role expectations, allowing the identity to manifest; when others who observe such behaviors grant the individual’s this identity they claim, the individual can internalize the identity and interpret it as self-defining (Bartel and Dutton, 2001; Gomes and Felix, 2019). Therefore, similar to the notion of calling enactment (Conway et al., 2015; Lysova and Khapova, 2018), there is an established assumption that an identity must be enacted to be self-descriptive (Ibarra, 1999; DeRue et al., 2009). Therefore, identities not yet or no longer held are not considered self-defining because they are not enacted (Obodaru, 2012; Strauss et al., 2012). Past, possible, ideal, ought, and alternative selves are traditionally defined as self-comparisons and not self-definitions (Ashmore and Jussim, 1997). This understanding explains why scholars posit that professional identities must be enacted through activities in a formal work role (Ramarajan and Reid, 2013; Petriglieri, 2015).

The identity literature has developed beyond this narrow view of identity enactment. For example, Riach and Loretto (2009) suggested that retired, disabled, and unemployed individuals can describe themselves based on their currently enacted professional identities and also their past professional identities. Ramarajan and Reid (2013) proposed that identities not related to formal work roles are often negotiated and enacted in the work domain. Similar to Berg et al.’s (2010) study on unanswered callings, Vogel et al. (2016) proposed that employees whose values do not fit those of the companies they work for often enact non-work-related professional identities by adopting job and leisure crafting techniques. Obodaru (2017) suggested that individuals can compose a professional identity based on self-definitions enacted in a formal work role and also forgone professional identities enacted outside their formal jobs. These findings serve as sensitizing concepts to expand the notion of calling enactment.

Based on these theoretical advances, we suggest that calling enactment may not be restricted to actions related to a formal work role, as is taken for granted in the calling literature (e.g., Conway et al., 2015; Lysova and Khapova, 2018); instead, we believe that unanswered callings can be enacted in other ways. This opens new possibilities to explore how individuals can actively buffer the negative consequences of being unable to enact a calling in a formal work role.

### Calling Enactment and Workplace Personalization

So far, we have sustained that individuals can enact unanswered callings even when they cannot express these callings in their formal work role; however, expressing such identity-related callings through coping strategies, such as job and leisure crafting, is not always an easy task because it can often be at odds with expectations associated with a current formal occupation (Berg et al., 2010). In such situations, the use of symbolic communications at work allows individuals to express their identity-related callings without exposing themselves explicitly (Pratt and Rafaeli, 2001), as it is the case with job and leisure crafting.

The literature on symbols at work suggests that 70–90% of employees personalize their workstations (Wells and Thelen, 2002), and by doing so, they reveal their interests, passions, and identities (Elsbach, 2004). In this paper, we explore how people use workplace personalization – i.e., an employees’ active symbolic decoration or physical adjustment of a work environment (Byron and Laurence, 2015) – to enact their unanswered callings. Although symbols are physical manifestations that often entail greater meaning, for example, regarding one’s identity or calling (e.g., Gioia, 1986; Sundstrom and Sundstrom, 1986; Rafaeli and Worline, 2000; Gosling et al., 2002), we did not find studies that discussed workplace personalization in the context of unanswered callings. Notably, the research on the use of objects to represent the self is not new (e.g., Csikszentmihalyi and Rochberg-Halton, 1981; Wicklund and Gollwitzer, 1982). Among the many topics found in the literature on workplace personalization, we emphasize three aspects: relationships, self-regulation, and emotions.

Workplace personalization might enable the enactment of certain callings and identities through social interactions (Pratt and Rafaeli, 2001). The literature on symbols has highlighted their role in social exchanges; symbols are often used to communicate aspects of the self to others because they offer cues on elements important to the identity and potentialize opportunities for connections with others through shared interests. Therefore, symbols improve the chances of having successful interactions (Byron and Laurence, 2015).

Symbols used in workplace personalization also play a role in self-regulation, as they can prime attention and stimulate behavior toward achieving goals (Wicklund and Gollwitzer, 1982; Metcalfe and Mischel, 1999; Carver and Scheier, 2011). Because this study explores responses to unanswered callings, this notion is useful; symbols may help individuals keeping faith that they will realize past and future goals associated with their callings.

Finally, symbols evoke and express emotions. The meaning associated with symbols affects how people handle stressful situations (Gross, 1998). For example, Rafaeli et al. (1997) observed that organizational dress allowed employees to find comfort and relieve emotional tension as they performed their work roles.

After reviewing the literature on unanswered callings and presenting the sensitizing concepts derived from the literatures on identity and symbols at work, we introduce the research questions that oriented our grounded theory.

RQ1: How do individuals with unanswered callings utilize workplace personalization to represent these callings?
RQ2: How can workplace personalization help these individuals cope with the negative consequences of unanswered callings?

## Materials and Methods

To explore how individuals enact their unanswered callings through workplace personalization, we conducted a qualitative research, applying the principles of grounded theory (Glaser and Strauss, 1967). This methodology is performed through iterative waves of data collection and analysis, aiming to explain the phenomenon through a theoretical model that “grabs and fits the data” (Glaser and Strauss, 1967, p. 56). First, an initial sample of participants is selected and data are collected according to an initial research protocol, created based on sensitizing concepts from the literature. Next, when the data are analyzed, memos and initial codes generated must lead researchers to ask themselves what new questions can be posed to better explore the phenomenon, and what are the characteristics of research participants that should be selected in a second wave of data collection. These characteristics must provide sufficient variation and diverse perspectives so that the resulting grounded theory can encompass all the nuances of the phenomenon.

This process of selecting interviewees is called theoretical sampling. The new data are again analyzed to generate new codes and new adjustments to the research protocol. After the initial waves of data collection and analysis, the initial memos and first-order codes base the development of second-order codes and, subsequently, aggregate dimensions. Although the initial codes are more descriptive, the consecutive codes and aggregate dimensions are more analytical. This iterative process is complete when new interviews fail to reveal new relevant memos, codes, or dimensions (theoretical saturation).

In our study, we conducted iterative waves of data collection and analysis that amount to 47 interviews and non-participant observations. Participants in our study were employed by 32 organizations. The section “Initial and Expanded Samples” describes the process of data collection and analysis.

### Initial and Expanded Samples

Our first wave of data collection encompassed 15 employees from a large public organization: the Federal Court of Accounts. In Brazil, the public sector is known for selecting employees based on highly competitive knowledge-based tests that require months or years of preparation. The occupational background of individuals who are hired in these processes are often disconnected from the jobs they perform in the public sector. Therefore, we believed that many individuals driven to occupations unrelated to their present jobs would be found in these organizations.

Participants were mostly males, middle managers, had positions with high task autonomy, and had a preference to set clear barriers between their work and home lives. Therefore, in our second wave of data collection, we sought to diversify our sample so as to include individuals in diverse occupations, who worked in different types of organizations, with distinct levels of job autonomy, and with distinct preferences regarding the boundaries between work and home domains (Felix et al., 2018). Additionally, we also interviewed individuals who worked in shared and private workspaces, in organizations with distinct policies regarding workplace personalization, and in work settings frequented by customers. Because we aimed to find participants that could fulfill these criteria, we relied on snowball sampling to identify potential interviewees. The initial 15 participants referred the researchers to another 36 interviewees, who also referred the researchers to 68 additional participants: a total of 119 individuals were contacted.

### Participant Selection

Within our pool of 119 potential participants, we searched for those with unanswered callings and personalized workspaces. To do so, we followed the same procedure applied by Berg et al. (2010): we identified participants with unanswered callings by asking questions derived from the work of Wrzesniewski et al. (1997) on work orientation as callings, jobs, and careers. We asked participants to evaluate the extent to which statements expressing these three orientations accurately described their feelings about their current occupations: (a) work as enjoyable, meaningful, and as part of their identity (calling orientation); (b) work as a means to financially support themselves, their relatives, and their leisure time (job orientation); and (c) work as a means to achieve promotion, status, and challenge (carrier orientation).

Next, based on the definition of unanswered callings (Berg et al., 2010), we asked participants if they had any occupation other than their current formal work role that they felt driven to pursue. For each occupation mentioned, we followed up with this question: “Imagine you are working as a [occupation mentioned]. How similar would you say that doing such work would be to [the calling statement] to you? Why?” This allowed us to probe if the participants’ perceived these occupations to be intrinsically enjoyable and meaningful and a central part of their identity; thus, fulfilling the criteria to be considered an unanswered calling. In our poll of participants, 89 out of 119 (75%) fit the criteria for having unanswered callings.

In the next step, the researchers visited the workplace of the 89 participants and took a picture of their workstations from the perspective of someone entering the area. Then, based on Wells’s (2000) categories of workplace personalization, we classified the inventory of symbols observed in the pictures into 16 categories: (1) photographs, (2) paintings, (3) posters, (4) sculptures, (5) other art pieces, (6) paperweights, (7) news/cartoon clippings, (8) calendars, (9) plants, (10) diplomas, (11) certificates, (12) plaques, (13) children drawings, (14) bumper stickers, (15) toys/figures, and (16) cups.

According to the literature, certain coping strategies for dealing with an unanswered calling, such as job and leisure crafting, can offer emotional and practical risks for the individuals who adopt them (Berg et al., 2010; Obodaru, 2017). Nevertheless, symbols can be an effective way to establish communications while minimizing inter- and intrapersonal risks (Byron and Laurence, 2015). Therefore, we openly addressed the participants, asking about the symbols displayed in their workspaces. Forty-seven individuals offered explanations that alluded to their unanswered callings. These 47 participants comprise our final sample in this study. Their age ranged from 27 to 61 years old, 29 were males and 18 females. While some of them work as auditors and in the financial sector given our first round of data collection, we also encountered professors, psychotherapist, and engineers with unanswered callings. Table 1 presents demographic information about each participant, as well as their current occupations and unanswered callings.

**TABLE 1:** Research participants.

**Code**	**Age**	**Gender**	**Current occupation**	**Unanswered calling**	**Code**	**Age**	**Gender**	**Current occupation**	**Unanswered calling**
E1	29	Male	HR analyst	Consultant	E25	33	Female	Marketing manager	Ballerina
E2	36	Female	Secretary	Lawyer	E26	47	Female	Entrepreneur	Academic
E3	55	Female	Auditor	Yoga instructor	E27	39	Male	Postman	Actor
E4	42	Male	Accounting analyst	Comedy actor	E28	28	Male	Auditor	Musician
E5	40	Male	Auditor	Biologist	E29	38	Male	Computer programmer	Airplane pilot
E6	38	Male	Auditor	Ministry	E30	51	Male	Accounting professor	Psychologist
E7	37	Male	Auditor	Environmental engineer	E31	39	Male	Professor	Judge
E8	61	Male	Auditor	Doctor	E32	30	Female	Fashion designer	Musician
E9	32	Female	Auditor	Professor of philosophy	E33	33	Female	Marketing analysist	Fashion designer
E10	35	Male	Auditor	Musician	E34	29	Female	Receptionist	Teacher
E11	42	Male	Auditor	Photographer	E35	52	Male	Financial controller	Writer
E12	36	Female	IT director	Special education – teacher	E36	50	Male	Brand manager	Chef
E13	43	Female	Hospital manager	Personal trainer	E37	47	Male	Technology manager	Biologist
E14	46	Male	Auditor	Soccer player	E38	39	Female	Teacher	Ballerina
E15	45	Male	Sales manager	Personal trainer	E39	40	Female	Secretary	Yoga instructor
E16	35	Male	Professor	Designer	E40	34	Female	Marketing analyst	Sociologist
E17	46	Female	Nurse	Doctor	E41	3	Male	Bank clerk	Economist
E18	38	Male	Psychologist	Doctor	E42	48	Male	HR manager	Computer programmer
E19	27	Male	Bank manager	Agronomist	E43	30	Female	Psychoterapist	Fashion designer
E20	29	Male	Bank manager	Computer programmer	E44	45	Male	Professor	Ministry
E21	55	Male	Engineer	Table tennis player	E45	30	Female	Secretary	Physical educator
E22	41	Female	Salesperson	Special education – teacher	E46	37	Male	Professor	Musician
E23	38	Female	Lawyer	Veterinary	E47	36	Male	Logistics manager	Chef
E24	27	Male	Translator	Psychoterapist					

After verifying if a participant matched our criteria, we conducted in-depth, semi-structured interviews. We interviewed participants until we reached data saturation. This occurred after we completed the 42 interview. The interviews lasted from 26 to 85 min, with an average of 41 min, and were recorded and transcribed. All procedures performed involving participants were in accordance with the ethical standards of the institutional research committee of Fucape Business School, and an informed consent was obtained from all participants.

### Interview Protocol

The interview started with general questions about the participants’ career trajectories. Next, we asked questions about an alternative life in which their unanswered calling could have been answered, requesting participants to imagine and describe it in detail. We then asked if and how they represented this alternative life in their workplace personalization, and why they did so. After that, we asked questions to verify how this personalization influenced their feelings about the unanswered calling. Based on the principles of grounded theory, we maintained a flexible interview protocol, which we adjusted during the iterative process of data collection and analysis. Table 2 presents the consolidated interview protocol.

**TABLE 2:** Interview protocol.

**Sample questions**
How was your career trajectory so far?
Do you have an image of what your life might have become if something representing an important turning point in your past had happened in a different way? Please describe this alternative life in as much detail as you can. How would you, as a person, be in that alternative life?
Does the fact that you do not work as a [occupation] generate any negative feelings for you?
If somebody wanted to know you, do you think that it would be important that person know this alternative life?
Do you talk to other people about this alternative life? If yes, who are the people that you talk to and how they reacted?
What do the items in your workplace say about this alternative life?
What do the items in your workplace say about the occupation that you didn’t pursue?
Why do you display them?
How do you think that the act of displaying them influences your feelings about your unanswered callings?
How do you see your career in the future?
Do you think that these objects somehow lower any discomfort that you may have regarding your unanswered calling(s)? If so, how?
How satisfied are you with your life and your work nowadays? Is it different from any other moment of your life when you didn’t display these objects?

### Data Analysis

**FIGURE 1:**
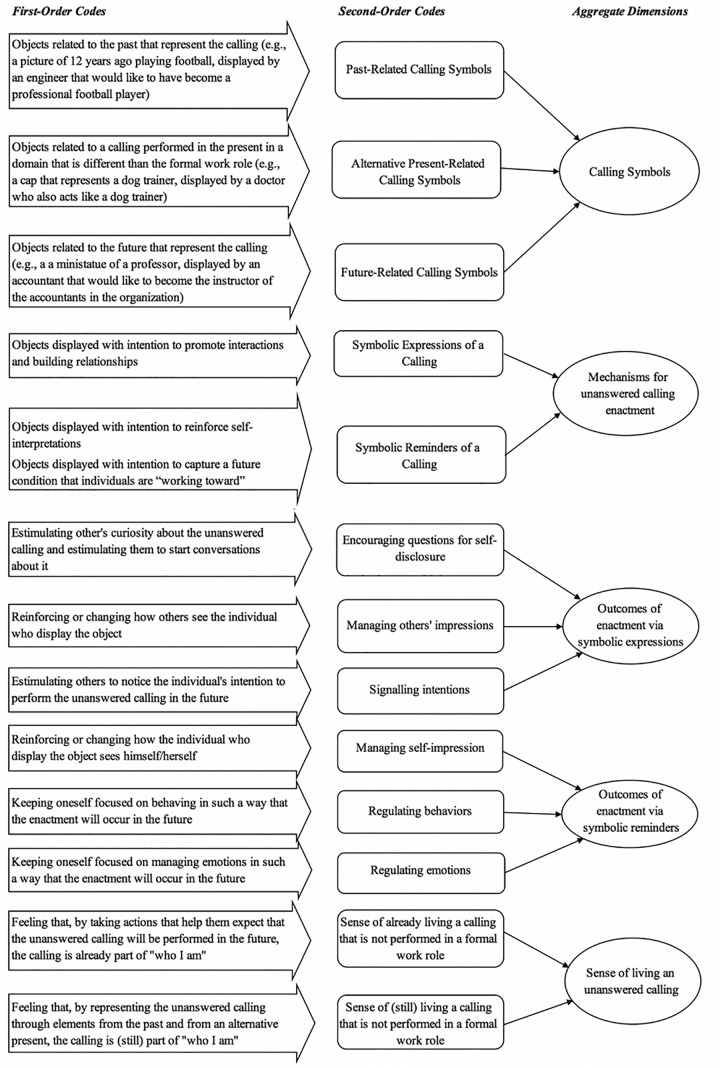
Data structure.

We started by developing broad memos, identifying relevant excerpts in their narratives (e.g., “trying to start conversations about an unanswered calling”). Next, as evidence with similar meanings emerged, we generated first-order codes, i.e., codes still close to the participants’ narrative (e.g., “stimulating others to notice the individual’s intention to perform the unanswered calling in the future”). Applying the principle of constant comparison, we analyzed the diverse first-order codes. As our analysis evolved and patterns of meaning became clear, we grouped those under second-order codes (e.g., “managing others’ impressions”). Based on the second-order codes, we identified emerging dimensions (e.g., “outcomes of enactment via symbolic expression”), which were then connected to each other in our model. Figure 1 summarizes the data structure, including first-order codes, second-order codes, and aggregate dimensions.

## Results

In this section, we present our answers to the paper’s two research questions. We found that when individuals personalize their workspace to represent their unanswered callings, they use objects that speak to their connection with those callings in the past (*past-related calling symbols*), in an alternative present, out of the formal work role domain (*alternative present-related calling symbols*), and in a desired future (*future-related calling symbols*).

When describing some of these objects in their workspace, our participants reported that the items allowed them to express their unanswered callings to others (*symbolic expressions of a calling*). This way, calling symbols help them start conversations about their callings (*self-disclosure*), influence the impression that other people have of them (*managing other’s impressions*), and show others that they intended to perform the calling in a formal work role in the future (*signalizing intentions*).

**FIGURE 2:**
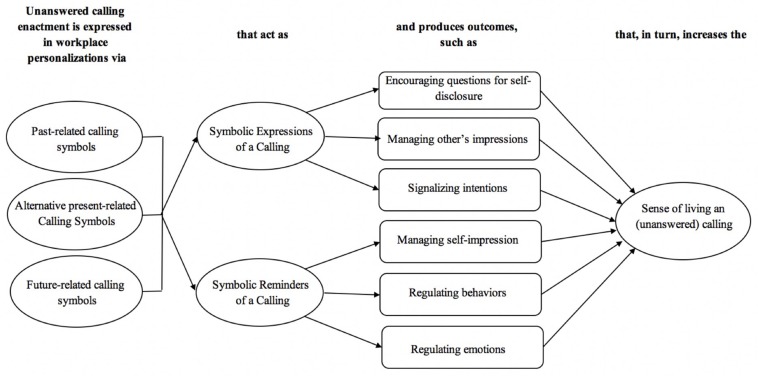
A model for unanswered calling enactment through workplace personalization.

In addition, calling symbols also allowed them to keep their unanswered callings in mind (*symbolic reminders of a calling*). In such situations, these objects help them retain aspects associated with the calling in their self-concept (*managing self-impressions*), maintain their focus and control over their behaviors (*regulating behaviors*), and keep their emotions in check (*regulating emotions*). According to our interviewees, through the use of symbols both mechanisms, expressing a calling (an interpersonal process) and reminding a calling (an intrapersonal process), help them buffer the negative consequences of not performing a calling formally and make them feel as if still (or already) living their unanswered callings (*sense of living an unanswered calling*). Figure 2 summarizes these findings. Each dimension is further described in the next sections.

### Representing Unanswered Callings Through Workplace Personalization

In our first research question, we asked how individuals utilized workplace personalization to represent their unanswered callings. Our findings show that such callings are represented symbolically by displaying personal objects in the workplace, in positions that can be visible to others, or not. We use the term “calling symbols” to refer to the physical objects that individuals display to communicate meanings associated with a calling-related occupation. We classified the calling symbols in three categories: past-, alternative present-, and future-related calling symbols.

#### Past-Related Calling Symbols

We called past-related calling symbols the objects displayed that represented a calling performed in the past. These calling symbols are displayed to express one aspect of the self that was actively enacted in some moment of their lives but is not performed in a formal work role currently. For instance, E23 displayed a picture of her helping dogs (unanswered calling: veterinary), E41 a statue that represented an economist (unanswered calling: economist), and E15 one diploma (unanswered calling: personal trainer). All these symbols were related to past selves that our research participants desired to keep alive.

“This is a picture of the times I used to play soccer. (…) I haven’t played for a long time, my knee doesn’t let me. But it is a way for me to keep this side of me alive. Without something like that, the memory fades and then the knee injury gets even harder to deal with” (E14, male, auditor, unanswered calling: soccer player).

By portraying this object, this participant “keeps this side [of his identity] (…) alive.” The traditional view in identity theory would not consider this representation as self-defining because the calling-related identity is no longer enacted formally (Strauss et al., 2012). Similarly, according to the calling literature and current assertions of the term “calling enactment” (Conway et al., 2015; Lysova and Khapova, 2018), the use of calling symbols would not be conceived as a calling enactment, because the individual no longer performs these callings in formal work roles. Notably, and in line with the idea that individuals can describe themselves based on both currently enacted professional identities and past professional identities (Riach and Loretto, 2009), we believe that calling symbols associated with past identities allow individuals to enact their unanswered callings.

#### Alternative Present-Related Calling Symbols

Some participants also reported that their calling symbols were associated with callings performed in the present but not in their current work role. In this case, objects referred to hobbies that are self-defining and meaningful to them. For example, we found a mini statue of a guitar (E10, unanswered calling: musician), a reiki magnet (E39, unanswered calling: yoga instructor), and a cup with a printed image of a photographer (E11, unanswered calling: photographer). Referring to the latter, the participant explained:

“I am not a professional photographer, but I like to be seen as if I were. I confess that when I got this cup as a present, I decided to bring it to my workspace because I wanted people to see me as a photographer. It helps me see myself as a photographer as well” (E11, male, auditor, unanswered calling: photographer).

By showing that cup to others in his workplace, this participant enacted a forgone identity-related calling, seeking an external validation for how he sees himself. Although he acts as an amateur photographer, being recognized as such by individuals with whom he has a professional relationship helps him retain his calling in his self-definition. This finding is in line with recent developments from identity research, which indicates that identities that are not part of a formal work role are often enacted in the work domain (Ramarajan and Reid, 2013). Symbols that represent a calling performed in the present in an alternative domain are also a means of enacting an unanswered calling.

#### Future-Related Calling Symbols

Some objects we found in our study did not represent a calling that was performed in the past or in an alternative present. They represented a calling the participants intended to perform professionally in the future. Based on the mainstream calling literature, we could affirm that those objects simply represent callings that individuals want to enact in the future; however, although the calling represented by those objects can be performed in the future, the display of such objects also allows the individuals to already enact that calling in the present. This occurs because future-related calling symbols keep an individual’s orientation toward a calling active and allow them to see themselves as “already becoming” who they wish to be. The future-related calling symbols that we encountered included objects such as a Nietzsche mini statue (E9, unanswered calling: professor of philosophy), a crystal (E3, unanswered calling: yoga instructor), and a mini globe (E29, unanswered calling: airplane pilot).

“This globe represents my dream of, someday, become an airplane pilot. I feel this is what I am called to be (…). When I look at this globe, I see that aspect of myself there. Although I don’t know if I will become an airplane pilot, it is like keeping the dream alive. It makes me already see myself in the process of becoming an airplane pilot” (E29, male, unanswered calling: airplane pilot).

The process observed in this case, regarding a future-related calling symbol, allows us to expand the theory on calling enactment. When this man says that he already sees himself “in the process of becoming an airplane pilot,” even though he is not working as such, he is “acting out” the unanswered calling. In this case, representing future desired states through workplace personalization seems to be a means to live an identity not yet performed in a formal work role. This observation broadens our present understanding of how callings can be enacted.

All these strategies used to represent the unanswered calling symbolically allow these individuals to express an alternative and meaningful aspect of their identity. In addition, participants also used calling symbols because they feel it is safe: “speaking openly about these things [their unanswered callings] could put me in danger because sometimes it is not so good to be seen as someone that has a different professional dream” (E3). According to our interviewees, others could see them as “frustrated,” “without focus,” or “unfit” for their current activities. Thus, as workplace personalization allows them to expose their unanswered callings to fewer individuals and function as a safe and effective way to enact the unanswered calling. The section “Buffering the Negative Consequences of Not Living a Calling” more clearly explains the outcomes of calling enactments through workplace personalization and how they can ease the negative consequences of an unanswered calling.

### Buffering the Negative Consequences of Not Living a Calling

Our second research question explored how workplace personalization can ease the negative consequences of not living a calling. The literature has suggested that trying to manifest an unanswered calling at work is not a risk-free activity; sometimes it can negatively affect how others think about an individual’s career or even incite feelings of stress and regret over forgone accomplishments related to their unanswered callings (Berg et al., 2010). Our results showed that workplace personalization functions as a coping strategy that minimizes such risks and that gives rise to both, interpersonal processes that allow *symbolic expressions of a calling* and intrapersonal processes that entail *symbolic reminders of a calling*.

#### Symbolic Expressions of a Calling

Many interviewees reported that by personalizing their workspaces with objects that represented distinctive characteristics of themselves, they developed interactions and relationships that allowed them to retain their unanswered calling as part of their identity. As most of the literature on symbols at work has discussed how physical manifestations are used to communicate meanings (Byron and Laurence, 2015), the emergence of this mechanism was expected. We observed three outcomes that unfold from such symbolic expressions of a calling: “*encouraging questions for self-disclosure*,” “*managing others’ impressions*,” and “*signalizing intentions*.”

##### Encouraging questions for self-disclosure

According to our interviewees, calling symbols encouraged conversations in which they could reveal aspects of their unanswered callings unknown to others. In the following case, a man who displayed a diver diploma told us that this calling symbol, which relates to an alternative present, helped him generate curiosity in others, and provoke conversations about this hobby, giving him the opportunity to talk about his unanswered calling, to be a biologist, and to feel more comfortable with who he is.

“I hang this poster for people to know of my interest so that they come to me and ask why I took the course, what my motivation was. When that happens, I explain why I took the course, that I like to dive and that this is a way of dealing with this calling I have to be a marine biologist and this wish to preserve nature. I like to be seen like that, it makes me feel more authentic” (E5, male, unanswered calling: biologist).

Our results show that this process is enacted effortlessly, especially when symbols represent unanswered callings that are not seen as potentially in conflict with the current jobs. However, when this potential conflict exists, our participants affirmed that they managed the degree of self-disclosure when answering questions. When they believed that disclosing the unanswered calling to a specific person could threaten their careers, they tended to provide more evasive answers and “pretend that it is not as important as it is” [E18]. Thus, this process is a coping strategy that allows individuals to manage risk when revealing those hidden aspects of themselves and which also gives rise to a sense of living an unanswered calling, even though this is not their formal work.

##### Managing others’ impressions

Some participants also reported that displaying calling symbols allowed them to manage others’ impressions about them. This was more common among those with socially desirable unanswered callings. By displaying a calling symbol, they influenced how others in the workplace saw them, as illustrated by the following account:

“This picture is on my desk because I like to be seen as a fashion designer, although I’m not. We work at a marketing agency; everyone is connected to the world of creation, but I like it when they see this picture because it makes me see myself as someone different. It improves my self-esteem” (E33, female, unanswered calling: fashion designer).

A notable aspect of this process is the risk of producing undesired results if the calling is, to some degree, not socially accepted or considered by others to be too distinctive or in conflict with the current job. To minimize this risk, some participants said that they used symbols that tend to be recognized and valued only by those who also have some familiarity and interest in the calling. For example, one man, who previously worked as a therapist, displayed a reiki symbol (a form of alternative medicine) on his desk. This participant said that most people who know that symbol have a positive attitude about what it represents and that it makes him comfortable with that object. Thus, when someone asks him about his connection with that past-related symbol, the conversation that unfolds is usually positive and results in external validation of that calling, making him feel that he is indirectly living the unanswered calling.

##### Signalizing intentions

Some participants also reported that calling symbols helped them communicate their intention to enact the unanswered calling in the future. This strategy was used when they wanted to make a career change inside the organization but felt uncomfortable discussing this intention openly, due to the risk of being perceived as less committed to their current job. By displaying a calling symbol, they could express their desire to include aspects of the unanswered calling in their formal work role or to be reallocated to a different position. In other words, it is a “more implicit way to show what I want to do without having to say it openly and run unnecessary risks” [E40]. The following quote illustrates this strategy as enacted by a participant using a future-related calling symbol:

“I work here at the bank in the large accounts section, which includes several clients from here, the more urban area of the city. But I hang this work of art that represents a wheat field as a way to signal my vocation to the rural area, which is where I came from as a child and where I think I could contribute better. The people who can relocate me to this area pass by and stare. I prefer to communicate it this way rather than to speak directly, as it might sound like a lack of interest in the urban area. So, I keep going. I’ve given my message, and I cannot be more explicit than that” (E19, male, unanswered calling: agronomist).

Although we presented these three outcomes of expressing a calling separately, they are not mutually exclusive. Some interviewees mentioned two or three of them when talking about their calling symbols. These three processes helped these individuals to develop a sense that they are living their callings. The expression of a calling was the most frequent account for the use of workplace personalization as calling symbols. Nevertheless, we also found evidence that such symbols can act as reminders of a calling, as described next.

#### Symbolic Reminders of a Calling

Although the literature on workplace personalization has portrayed this concept as a means to communicate information about “who someone is” to others (Sundstrom and Sundstrom, 1986), we observed that calling symbols are also displayed for “personal consumption.” Some calling symbols were discernible to nobody except the interviewees. In addition, they were positioned out of immediate view, suggesting that these objects were not meant for communicative use (to be seen by others), but for personal use (Byron and Laurence, 2015). Gosling et al. (2002) also found evidence that some workplace symbols are intended for the self, which they called “self-directed identity claims.”

In our study, this type of personalization reinforces self-interpretations already held (past- and alternative present-related calling symbols) or entail a future position that individuals are “working toward” (future-related calling symbols). Therefore, when calling symbols help individuals remember callings related to past professional lives or to what they do in their leisure time that relates to a calling not performed professionally, they allow them to reconnect with these aspects of their self-concepts, of who they “used to be” or who they “are.” Future-related calling symbols also help individuals reinforcing their self-interpretations, by reminding them of who they “are going to be someday.” Such intrapersonal processes help them retain the unanswered calling as a part of their identity and increase the sense of living the unanswered calling. We observed three outcomes that unfold from the use of such symbolic reminders of a calling: “*managing self-impressions*,” “*regulating behaviors*,” and “*regulating emotions*.”

##### Managing self-impression

Some participants reported that they displayed symbols at their workstation to make them reinforce or change how they perceived themselves. By keeping these objects, they could align their impression of themselves with the person they would be if they performed their unanswered calling in a formal work role. Thus, some participants customized their work stations to influence their self-impression and connect to present selves and past selves, as well as with future selves that they nurtured the intention to become. For example, a participant whose unanswered calling was to be an environmental engineer – and who had become professionally involved with environmental causes early in his career – displayed a globe paperweight on his desk. Although he had never been an environmental engineer, that past-related calling symbol allowed him to think of himself as an engaged person, who had made contributions to the preservation of the planet’s environmental condition, and therefore to retain this aspect of his unanswered calling in his definition of “who he is.” This, in turn, helped him develop a sense of living the calling in his formal work role.

“I’ve never actually worked as an environmental engineer. But I spent a lot of time involved in activism, contributing in one way or another. So, I feel like a defender of the planet. Today, my job has nothing to do with the issues that move me, but I bought this globe, and I leave it here for me not for others – because no one knows about this – but for myself because I look at it, and I remember who I am, who I have been, the things I have already done” (E7, male, unanswered calling: environment engineer).

Our study revealed that workplace personalization also promoted self-regulatory processes, which include the regulation of action and affect (Carver and Scheier, 2011). We found evidence that some interviewees used symbols to enact their unanswered callings because it helped them change their behaviors and managing their emotions by remaining focused on their goals; these outcomes involve regulating behaviors and regulating emotions.

##### Regulating behaviors

Although the enactment of a calling is generally viewed as dependent on actions performed in a formal work role, we found evidence that they can also be enacted by using future-related calling symbols, since they stimulate some individuals to remain focused on the construction of a future self, when the calling is answered. One participant, whose unanswered calling was to teach children with special needs, displayed a sheet of paper on her workstation with a child’s handprints. Those are not her daughter’s hands, as many suppose, but the hands of a child with Down syndrome. According to the participant, when she looks at that symbol, she remembers her intention to change occupations in the future; it also reminds her that in order to live her unanswered calling she must continue to work as a cosmetics saleswoman until she can make this transition safely. While this increases her confidence that the unanswered calling will be enacted in a formal work role in the future, it also helps her regulating her present behavior, since it allows her to perform her present work better.

“My calling is to work with children with special needs, but I could not do it for several reasons. The problem is that I need to financially prepare myself to make this transition because a salary in this area would not be enough for me to support my family. So, these little hands here by my side help me keep my energy high, because I know I must endure the things I do not like here so that I can afford my professional dream one day. (…) Nobody knows this, they think it’s my daughter’s hands, but that’s okay, because it’s something for myself (…) It makes me know that this situation is temporary and it helps me deal better with my frustration” (E22, female, unanswered calling: special education – teacher).

##### Regulating emotions

Calling symbols also help individuals regulating their emotions. We found that interviewees used symbolic reminders of a calling to achieve situation modification, an emotion regulation tactic that entails specifically altering one’s physical environment to change the emotional impact of a stressful situation (Gross, 1998). Some participants reported that they displayed future-related calling symbols not directly associated with their unanswered callings but related to a desired emotional state, which would help them handle the stress experienced in the current work role. Such symbols reminded them that they were creating the conditions that would allow them to live their callings in the future. This interpretation was based on accounts such as the following:

“Working with customer service here is not easy. I’m always listening to complaints, and I’m even called names. This origami reminds me of the Japanese culture, of keeping the balance, being in control of yourself. I need this job to make some money because they pay well, despite being a very unpleasant environment. So, when I’m stressed, I look at the origami, and I remember who I want to be, that I still want to open a physical education clinic and that this is the contribution I want to make to the world. But to get there, I have to keep calm, avoid customer complaints and increase my compensation to save money and open my clinic in the future” (E45, female, unanswered calling: physical educator).

These three intrapersonal processes produced by symbolic reminders of unanswered callings allowed these individuals to increase their sense of living their unanswered callings through imagined selves. They made them see themselves as more connected to their callings and strengthened their beliefs that they can enact that calling formally, albeit in the future.

## Discussion

Although the literature has extensively explored the negative outcomes of not living a calling (Duffy et al., 2012; Gazica and Spector, 2015), we still know little about how people can alleviate the adverse consequences of having an unanswered calling. In this study, we explored how people enact unanswered callings through workplace personalization. Our findings suggest that people cope with unanswered callings not only through job and leisure crafting (Berg et al., 2010); they also cope with them through the use of calling symbols. Past, present, and future calling symbols allow the individual to retain the unanswered calling in their self-concept. This research contributes to the calling literature in four main ways.

First, we broaden the present understanding about calling enactments, by theorizing that callings can be enacted not only through work roles (Conway et al., 2015), but also through the use of calling symbols, which help expressing and remembering an unanswered calling. The role of symbolic self-representations at the workplace was not yet addressed in the calling literature. In this research, we observed that even when a calling is not answered in current formal work roles, some individuals influence how they think and feel about them by using symbols to act out their callings. Thus, our study shows that workplace personalization entails an important strategy for people to enact their unanswered callings and restore positive psychological states.

Second, the notion that calling symbols can forge a sense of living a calling is also relevant to the understanding of how people can achieve self-actualization in a world with so many career options. Duffy and Autin (2013) suggested that the positive outcomes of having a calling depend on living it and not perceiving it. Our results support that notion. However, a calling can be indirectly performed, or acted out, through symbolic action. Therefore, the positive outcomes of having a calling can still be experienced, even if people do not perform them in their current work roles, but if they manage to retain them in their self-concepts. This observation also challenges the understanding that an individual can only become a well-adjusted adult by forgetting non-lived alternatives (see also Obodaru, 2017).

Third, we observed that objects can be used to communicate meanings (Byron and Laurence, 2015). In this study, we followed Csikszentmihalyi and Rochberg-Halton’s (1981) advice to explore what goes between individuals and objects to understand people and what they can become. Objects that are calling symbols are used to communicate the unanswered calling to others and to oneself, thus producing specific positive outcomes through interpersonal and intrapersonal processes. By exploring the use of workplace personalization, we demonstrate that unanswered callings can be expressed not only through job and leisure crafting (Berg et al., 2010) but also through the use of such symbolic devices.

Fourth, our study adds to the literature about coping strategies regarding unanswered callings. Berg et al. (2010) have highlighted the risks in strategies such as job and leisure crafting, which can provoke negative feelings of stress and regret over forgone accomplishments related to unanswered callings. Workplace personalization is presented here as an alternative and fairly benign coping strategy, which seems to generate resignification, rather than stress (in the case of past- and alternative present-related calling symbols), and hope, rather than regret (in the case of future-related calling symbols). Calling symbols allow the enactment of unanswered callings without the unintended consequences observed in other coping strategies.

In addition to the calling literature, this paper also contributes to identity research, by broadening the understanding on how people enact identities that were forgone. The work of Obodaru (2017) introduced the notion that imagination regarding past and future selves are alternative forms of enactment for such identities. We observed that calling symbols also help individuals enacting their forgone identities, further expanding the notions that symbolic objects are used to represent past, present, and future selves at work (Byron and Laurence, 2015) while minimizing the risk of conflicts (Pratt and Rafaeli, 2001).

## Final Considerations

### Limitations and Future Research

This study has several limitations that leave open possibilities for future research. First, we have only explored workplace personalization as a domain in which calling enactment can be manifested. Therefore, we suggest that researchers explore symbolic strategies for calling enactment in other domains (e.g., attire, language, and social media). Second, our theory was developed through retrospective accounts. A longitudinal study can be performed to track individuals’ experiences from the moment they become aware of their unanswered callings and seek to understand how and when different coping strategies take place over time. Third, we found objects through which some participants represented their callings that were connected to activities performed by significant others. For example, a participant displayed a picture of her daughter dancing ballet to encourage questions for disclosing this aspect of her unanswered calling, to be a ballerina. Future research can explore the use of symbols to perform this type of vicarious enactment. Forth, although our research shows how workplace personalization can help people enact their unanswered callings, we didn’t explore how this strategy can be taught to individuals. Previous research (Asuero et al., 2014; Galla et al., 2015) discussed the role of education and training programs to cope with burnout and stress, including strategies from low-income countries (Paganelli et al., 2018). Future research can explore how interventions can also help people cope with the negative consequences of unanswered callings.

### Practical Implications

This study offers practical implications for leaders and individuals who want to better cope with their unanswered callings. Leaders who understand the relevance of their followers’ unanswered callings will have a greater ability to offer them conditions to better design their career development paths and encourage job and leisure crafting. They can read the signs of unanswered callings that their followers send through workplace personalization and then use this information. Individuals such as E12, the IT director whose accounts are quoted in this article’s epigraph, may also benefit from our findings. It is common for individuals to experience frustration and confusion when they realize they have an unanswered calling. In the process of handling this uncomfortable realization, many of them think that “moving on without looking back” is the only manner to deal with it. We suggest that even when individuals must give up living their callings in a formal work role, they are not obliged to abandon these aspects that define themselves. The use of calling symbols in workplace personalization as a coping strategy might help them retaining these calling-related self-defining elements, therefore soothing their discomfort.

## Conclusion

Our study revealed that when people enact an unanswered calling through workplace personalization, they can retain aspects of that calling in their self-concept and ease the negative consequences of not living it. In some cases, they could even enjoy some of the benefits of perceiving a calling without performing them in their formal work roles. This phenomenon occurs because workplace personalization can be used to represent unanswered callings they have performed in the past, informally perform in the present, and intend to perform in the future. The enactment of their unanswered callings through such calling symbols functions as a coping strategy. They give rise to interpersonal and intrapersonal processes that allow them to express and think about their callings, which buffers the negative consequences of not answering them.

## Data Availability

The datasets for this manuscript are not publicly available because we conducted personal interviews with our research participant. Some of them provided sensitive information and their voices could be recognized. For this reason, we didn’t give publicity to the interviews. Requests to access the datasets should be directed to bfelix@fucape.br.

## Ethics Statement

Ethics Committee: Programa de Pós Graduação em Administração/Fucape Business School. This study was carried out in accordance with the recommendations of the FBSR Guidelines, ethics committee at Fucape Business School with written informed consent from all subjects. All subjects gave written informed consent in accordance with the Declaration of Helsinki. The protocol was approved by the ethics committee at Fucape Business School.

## Author Contributions

BF has collected all the data. BF and FC analyzed the data and wrote the manuscript.

## Conflict of Interest Statement

The authors declare that the research was conducted in the absence of any commercial or financial relationships that could be construed as a potential conflict of interest.
